# Towards precision medicine: The application of omics technologies in asthma management

**DOI:** 10.12688/f1000research.14309.2

**Published:** 2018-05-18

**Authors:** Chiara Scelfo, Carla Galeone, Francesca Bertolini, Marco Caminati, Patrizia Ruggiero, Nicola Facciolongo, Francesco Menzella

**Affiliations:** 1 Department of Medical Specialties, Pneumology Unit, Arcispedale Santa Maria Nuova- IRCCS, Azienda USL di Reggio Emilia, Reggio Emilia, 42123, Italy; 2 Department of Bio and Health Informatics, Technical University of Denmark, Kongens Lyngby, 2800, Denmark; 3 Asthma Center and Allergy Unit, Verona University Hospital, Verona, 37134, Italy

**Keywords:** Severe asthma, Omics sciences, Inflammation, Precision medicine

## Abstract

Asthma is a chronic obstructive respiratory disease characterised by bronchial inflammation. Its biological and clinical features have been widely explored and a number of pharmacological treatments are currently available. Currently several aspects of asthma pathophysiological background remain unclear, and this is represent a limitation for the traditional asthma phenotype approach. In this scenario, the identification of new molecular and clinical biomarkers may be helpful in order to better understand the disease, define specific diagnostic tools and highlight relevant novel targets for pharmacological treatments. Omics technologies offer innovative research tools for addressing the above mentioned goals. However, there is still a lot to do both in the fields of basic research and in the clinical application. Recently, genome-wide association studies, microRNAs and proteomics are contributing to enrich the available data for the identification of new asthma biomarkers. A precise approach to the patient with asthma, particularly with severe uncontrolled asthma, requires new and specific therapeutic targets, but also proper tools able to drive the clinician in tailoring the treatment. On the other hand, there is a need of predictors to treatment’s response, particularly in the field of biological drugs, whose sustainability implies a correct and precise selection of the patients. Translating acquired omics knowledge in clinical practice may address the unmet needs described above, but large-scale studies are required in order to confirm their relevance and effectiveness in daily practice. Thus in our opinion the application of omics is still lagging in the real-life setting.

## Introduction

Several aspects of asthma heterogeneity both from a clinical and pathophysiological perspective remain still unclear. A number of treatment options have been developed over time, from the widely used corticosteroids to personalized approaches, including recently introduced biological therapies. The new classification of severe asthma is based on endotypes, whose definition relies on the features of the underlying inflammation. The endotypes define traditional phenotypes by describing their pathophysiological mechanisms ^1, 2^. Exploring endotypes and phenotypes require the identification of specific molecular targets, which can be addressed by precision treatments such as biologic drugs. The management of severe asthma is benefitting from personalized medicine approaches based on the characterization of an increasing number of endotypes, which represent the targets of specific therapies ^3^. These are mainly represented by the Th2-high subtype and the Th2-low subtype, characterized by the presence of eosinophilic or neutrophilic/paucigranulocytic airway inflammation respectively ^3^. Currently targeted therapies for a number of Th2 – low endotypes are still lacking ^4^. For this and other reasons, the need for increasing the effectiveness of personalized therapies opens the field to the omics approaches.

New asthma phenotyping has led to a growing interest in targeted therapies. The search for new pharmacological targets has caused interest in understanding the pathophysiological and molecular mechanisms underlying asthma. So far, the majority of the available drugs target the Th2-cytokine pathway ^5^.

### What else could be done for the management of severe asthma

**Figure 1.**
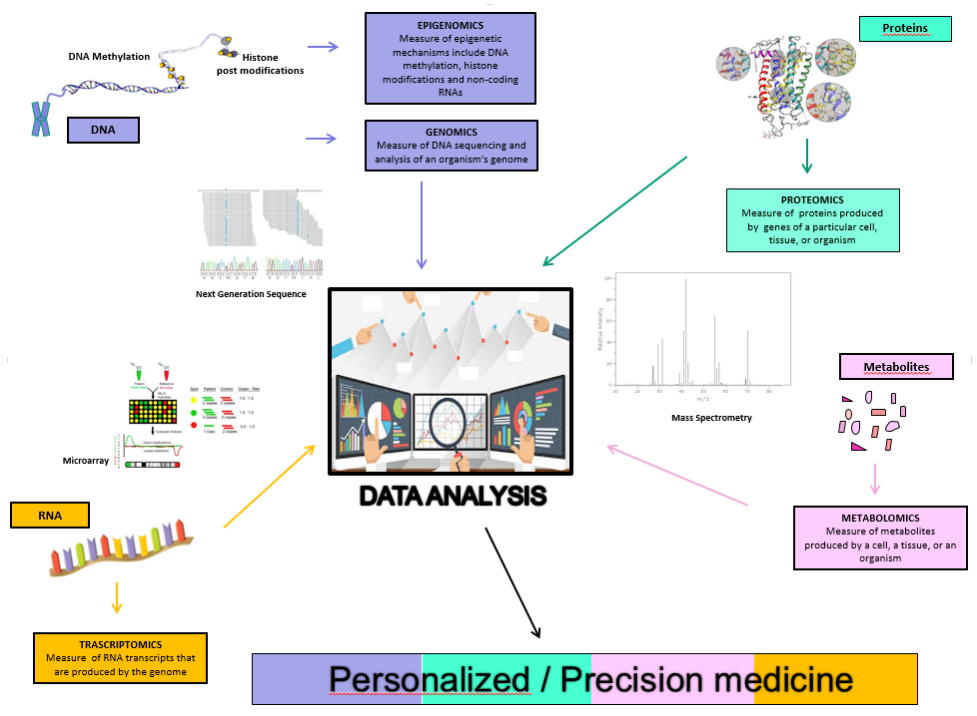
A systems biology approach: From omics science to precision medicine.

Omics technology supports precision medicine in identifying the most effective treatment for different clinical phenotypes, in contrast with the “one size fits all” approach. Indeed, omics sciences contribute to the definition of new biomarkers, which can be useful as hallmarks of a specific asthma endotype or phenotype, and relevant as novel targets for pharmacology treatments. In the field of molecular biology, omics is a neologism that indicates high-throughput experimental technologies providing the tools for comprehensively monitoring the disease processes at a molecular level. The suffix “ome” comes from “chromosome” and currently includes several biological fields such as genomics, transcriptomics, proteomics, metabolomics and epigenomics. Genomics and transcriptomics have been using to identify genes associated with asthma severity ( Figure 1). Recent genome-wide association studies (GWAS) have shed light on distinct pathways that contribute to asthma inflammation. Genes such as HLA, IL13, IL33, thymic stromal lymphopoietin (TSLP) involved in Th2 pathway, IL-1 receptor–like 1 (IL1RL1), encodes ST2, and the receptor for IL-33 are associated with asthma onset. In contrast, it is well-known that the risk of childhood asthma is associated with the 17q21 locus encoding the ORMDL3 and GSDML genes ^6, 7^. Transcriptomics are focused on the identification of an increasing number of several types of RNA with different function, e.g messenger RNAs (mRNAs) and long non coding RNAs (lncRNAs) but particular attention should be given to the investigation of microRNAs (miRNAs). MiRNAs are small non-coding single strand RNA chains involved in post-transcriptional regulation processes. MiRNAs play a key role in regulating cell functions as well as in modulating the inflammatory pathways. They may influence the single endotype profile in the complex asthma phenotype picture, therefore, the relevance of miRNA as a biomarker has been increasingly investigating. MiRNAs can be collected through peripheral blood sampling (Circulating miRNA), or, more invasively, through bronchial biopsies and induced sputum ^8, 9^. Circulating miRNA deserves a specific interest, because they might be a non-invasive biomarker useful in asthma diagnosis and characterisation, as demonstrated in a recent study. According to the authors, a specific subset of circulating miRNAs (miR-125b, miR-16, miR-299-5p, miR-126, miR-206, and miR-133b) was found in patients with allergic rhinitis and asthma ^10^. MiRNA 192 in peripheral blood was under-expressed in blood of asthmatic patients underwent an allergen inhalation challenge ^11^. Different levels of miR-1248 in serum of asthmatic vs non-asthmatic patients have been also documented. MiR-1248 is involved in the regulation of IL-5 pathway ^11^.

Epigenomics includes DNA methylation, histone modifications and non-coding RNAs previously described. Its role is to control gene expression acting on DNA structure. Several genes related to asthma are regulated by epigenetic mechanism such as genes involved in T-effector pathways (Interferon INF-γ, Ιnterleukin (IL)-4, IL-13 and IL-17), T-regulatory pathways (forkhead box P3 [FoxP3]) and airway inflammation (arginase [ARG]) ^12^. The “methylome” (the set of DNA methylation patterns) has been increasingly investigating through highly sophisticated sequence-based assays. Epigenetic mechanisms could lead to the identification of new asthma biomarkers. Recently, an epigenetic association between serum IgE concentration and methylation at different loci derived from DNA of leukocites has been described. Methylation at these CpG islands differed significantly in isolated eosinophils between subjects with and without asthma and high IgE levels ^13^.

Modern and advanced technologies, such as mass spectrometry, allow the detection of several proteins involved in the inflammatory mechanisms of asthma. Today several biomarkers can identify Th2-high endotypes (serum IgE, serum periostin, blood eosinophil, exhaled nitric oxide eNO, dipeptidyl peptidase 4 (DPP-4 serum) in sputum. Among the proteomics signatures characterized so far, Galectin-3 deserves to be mentioned. It was indeed demonstrated that this protein is expressed in omalizumab responders only. Furthermore, galectin–3 seems to be associated with a more evident improvement of respiratory function in asthmatic patients treated with omalizumab ^14^.

The increasing interest in metabolomics, is mainly due to its prospective clinical applications. Metabolomics could play an important role in measuring the concentrations of the metabolites generated in living system. According to recent findings it is possible to define the metabolic profile through different matrix including exhaled breath, urine, plasma and serum ^15, 16^, Exhaled breath condensate (EBC) is a promising tool for the detection of asthma biomarkers. This biological sample could be used as a natural matrix of the respiratory tract, carrying useful biomarkers which allow to monitor changes in inflammatory airways diseases ^17^. As recently demonstrated, electronic nose (eNose) and nuclear magnetic resonance (NMR)-based metabolomics could play a role in phenotyping chronic airway disease regardless of the diagnosis of asthma or COPD, suggesting therapeutical targets for a tailored respiratory medicine ^18^. EBC contains different inorganic molecular species such as nitric oxide (NO) and carbon monoxide (CO) and also volatile organic compounds. Currently a branch of metabolomics called “breathomics” focuses on VOCs from the respiratory tract. VOCs represent potential non-invasive metabolic biomarkers, particularly in the diagnosis and monitoring of pulmonary diseases including asthma ^19^. Moreover, an electronic nose able to discriminate asthmatic from healthy controls by detecting different VOCs in exhaled breath has been developed ^19, 20^. Therefore, metabolomics could play a key role in identifying biomarkers and improving asthma endotyping.

## Conclusion

The application of omics technology in asthma is following other research fields, such as oncology ^21^. Similarly, monoclonal antibodies (mAbs) for severe asthma have been recently introduced, while biological therapies addressing rheumatic diseases, solid tumors and blood cancer arrived more than a decade before. From 2006 to 2017 omalizumab was the only available treatment for severe allergic asthma. Only in recent years research and knowledge on new drugs has been developed to achieve new and more effective therapeutic options ^22^. Despite an increasing interest in omics technologies, none of the omics signatures mentioned above have been translated into clinical practice. We believe that there is an urgent need for large-scale studies. Particularly, specific Randomized Controlled Trials would be necessary to definitively confirm the clinical relevance of omics and reinforcing omics’ role in searching for new biomarkers and prognostic factors. The need for correctly selecting the right mAb for the right patient is one of the key points in severe asthma management. The real challenge for researchers and clinicians in the “omics era” is therefore translating acquired knowledge into clinical practice in order to emphasize omics’ role in precision medicine and to predict response to treatments. Unfortunately, in our opinion we are still far from that scenario.

## Data availability

The data referenced by this article are under copyright with the following copyright statement: Copyright: © 2018 Scelfo C et al.

Data associated with the article are available under the terms of the Creative Commons Zero "No rights reserved" data waiver (CC0 1.0 Public domain dedication). 

No data is associated with this article.
