# Supplementary material for: Towards precision medicine: The application of omics technologies in asthma management
Source: F1000Res. 2018 May 18;7:423. Originally published 2018 Apr 4. [Version 2] doi: 10.12688/f1000research.14309.2 (PMC5964625; doi:10.12688/f1000research.14309.2)
Supplement: Peer review history

# Peer review: Referee response for version 2

**DOI:** 10.5256/f1000research.16336.r34191

**Published:** 2018-05-22

**Authors:** Mauro Maniscalco

**Affiliations:** 1 Department of Respiratory Rehabilitation, ICS MAUGERI Institute of Telese Terme, Telese Terme, Italy

The authors have revised the manuscript. I have no further comments to make.

I have read this submission. I believe that I have an appropriate level of expertise to confirm that it is of an acceptable scientific standard.

# Peer review: Referee response for version 1

**DOI:** 10.5256/f1000research.15571.r33170

**Published:** 2018-05-10

**Authors:** Mauro Maniscalco

**Affiliations:** 1 Department of Respiratory Rehabilitation, ICS MAUGERI Institute of Telese Terme, Telese Terme, Italy

Bronchial asthma is a complex and heterogeneous pathology due to multiple mechanisms that are not present in all patients at any given time-point, or in the same patient at different time-points.

 Furthermore this disorder is not characterized by a single biomarker, but by a panel of biomarkers which describe its molecular aspects.

 In the last years, “omics” sciences have improved disease phenotyping by linking the molecular mechanisms to the clinical field.

 In their opinion article Scelfo and colleagues discussed about the application of omics sciences in the asthma characterization.

 The topic is very interesting although I have some concerns regarding some aspects of the article:

 Firstly, the main concept of omics sciences, such as epigenomics and metabolomics, should be explained in a more extensive manner.

 Furthermore, several other proteins other than galectin- 3 are associated to severe asthma. I suggest to include other examples.

 Other matrixes (please prefer matrix to sample) have been used to metabolomics analysis in asthma such as exhaled breath condensate. This part should be extended, considered recent findings on the possibility to phenotype asthmatic patients using this method.

I have read this submission. I believe that I have an appropriate level of expertise to confirm that it is of an acceptable scientific standard, however I have significant reservations, as outlined above.

# Peer review: Referee response for version 1

**DOI:** 10.5256/f1000research.15571.r32719

**Published:** 2018-04-24

**Authors:** Claudio Micheletto

**Affiliations:** 1 Respiratory Unit, Mater Salutis Hospital, Legnago, Italy

The heterogeneity and complexity of the asthma syndrome necessitates a different approach to its management. The use of clinical features and physiological and inflammatory data is no longer sufficient. Omics data and clustering will provide a greater chance of phenotyping asthma according to the mechanisms driving the disease in each phenotype, from which a composite set of biomarkers could be used to define and categorise the endotypes. This will help to develop personalised medicine for asthma that will allow for more precise treatment and also provide a source of novel targets and hence new treatments for each defined endotype. It is high time that personalised medicine be applied to the whole spectrum of asthma.

 The application proposed by the authors is very promising, because it allows a precise phonotyping of asthmatic patients and a correct choice of therapeutic agents, in particular as regards the treatment with biologicals.

I have read this submission. I believe that I have an appropriate level of expertise to confirm that it is of an acceptable scientific standard.

# Response

**Published:** 2018-05-08

**Authors:** Chiara Scelfo

**Affiliations:** Santa Maria Nuova Hospital at Reggio Emilia, Italy

Dear Dr. Micheletto,

We thank you for agreeing to review our manuscript. We also thank you for your valuable and positive comments. This is an incentive for us to try to improve our work.

My best regards,

Chiara Scelfo and coworkers
